# Papaverine Prevents Vasospasm by Regulation of Myosin Light Chain Phosphorylation and Actin Polymerization in Human Saphenous Vein

**DOI:** 10.1371/journal.pone.0154460

**Published:** 2016-05-02

**Authors:** Kyle M. Hocking, Gowthami Putumbaka, Eric S. Wise, Joyce Cheung-Flynn, Colleen M. Brophy, Padmini Komalavilas

**Affiliations:** 1 VA Tennessee Valley Healthcare System, Nashville, Tennessee, United States of America; 2 Department of Surgery, Vanderbilt University Medical Center, Nashville, Tennessee, United States of America; University of Minnesota, UNITED STATES

## Abstract

**Objective:**

Papaverine is used to prevent vasospasm in human saphenous veins (HSV) during vein graft preparation prior to implantation as a bypass conduit. Papaverine is a nonspecific inhibitor of phosphodiesterases, leading to increases in both intracellular cGMP and cAMP. We hypothesized that papaverine reduces force by decreasing intracellular calcium concentrations ([Ca^2+^]_i_) and myosin light chain phosphorylation, and increasing actin depolymerization via regulation of actin regulatory protein phosphorylation.

**Approach and Results:**

HSV was equilibrated in a muscle bath, pre-treated with 1 mM papaverine followed by 5 μM norepinephrine, and force along with [Ca^2+^]_i_ levels were concurrently measured. Filamentous actin (F-actin) level was measured by an *in vitro* actin assay. Tissue was snap frozen to measure myosin light chain and actin regulatory protein phosphorylation. Pre-treatment with papaverine completely inhibited norepinephrine-induced force generation, blocked increases in [Ca^2+^]_i_ and led to a decrease in the phosphorylation of myosin light chain. Papaverine pre-treatment also led to increased phosphorylation of the heat shock-related protein 20 (HSPB6) and the vasodilator stimulated phosphoprotein (VASP), as well as decreased filamentous actin (F-actin) levels suggesting depolymerization of actin.

**Conclusions:**

These results suggest that papaverine-induced force inhibition of HSV involves [Ca^2+^]_i_-mediated inhibition of myosin light chain phosphorylation and actin regulatory protein phosphorylation-mediated actin depolymerization. Thus, papaverine induces sustained inhibition of contraction of HSV by the modulation of both myosin cross-bridge formation and actin cytoskeletal dynamics and is a pharmacological alternative to high pressure distention to prevent vasospasm.

## Introduction

The human saphenous vein (HSV) is the most commonly used conduit for coronary artery bypass grafting (CABG). However, HSV is regarded as inferior to the internal mammary artery (IMA) because of 50% failure rate within 10 years compared with 5% failure for the IMA[1,2]. Saphenous vein grafts commonly develop vasospasm during harvest and this spasm is typically treated by distension of the conduit with a hand held syringe[3] or with pharmacologic approaches. Manual distension can result in supraphysiological intraluminal pressures (up to 800 mmHg) that injure the fragile endothelial monolayer[4]. Vasospasm results in reduced nitric oxide production, decreased thromboresistance, and may contribute to saphenous vein graft failure [5].

Effective pharmaceutical approaches to vasospasm in HSV include glyceryl trinitrate, verapamil, and papaverine[3]. Because sodium nitroprusside (SNP) and nicorandil have been demonstrated to be less effective at treating vasospasm in HSV than papaverine[6], treatment with papaverine *ex vivo* prior to implantation of the HSV has been the most commonly used approach for preventing vasospasm[6,7,8].

Papaverine is a non-selective phosphodiesterase inhibitor found in the opium poppy[8]. It has been shown to increase cGMP and cAMP in smooth muscle,[9] both of which induce vasorelaxation[10,11]. Traditional vasodilators like SNP function by donating exogenous nitric oxide which acts via guanylate cyclase to increase cGMP. Cyclic GMP activates cGMP-dependent protein kinase (PKG) and mediates relaxation by reducing [Ca^2+^]_i_ concentration through calcium regulatory proteins [12]. Cyclic GMP- mediated relaxation involves both a reduction of [Ca^2+^]_i_ and activation of myosin light chain phosphatase 1, which reduces the sensitivity of the contractile apparatus to intracellular calcium[13,14]. PKG-mediated reduction of [Ca^2+^]_i_ have been proposed to involve several mechanisms including activation of Ca^2+^ -ATPase in the plasma membrane and sarcoplasmic reticulum[15], activation of Ca^2+^-activated K^+^ channels[16], inhibition of actin-activated Mg^2+^-ATPase[17], inhibition of inositol 1,4,5-trisphosphate formation by inhibition of phospholipase C activation[18], inhibition of G protein coupling to phospholipase C[19,20,21] and inhibition of Ca^2+^ release by the sarcoplasmic reticulum[22]. Several proteins including Ca^2+^ -activated K^+^ channel [23], phospholamban [24], and type I inositol 1,4,5-trisphosphate receptor[25],[26] have been reported to be phosphorylated in response to PKG activation which may contribute to the reduction of [Ca^2+^]_i_. A decrease in intracellular [Ca^2+^]_i_ causes inactivation of myosin light chain kinase, dephosphorylation of myosin light chain by the myosin phosphatase, which causes a reduction in the sensitivity of the contractile apparatus to [Ca^2+^]_i_[18,27,28,29]. CyclicGMP/PKG activation regulates calcium sensitization partly by activating the myosin light chain phosphatase[28,30] and by affecting the Rho-dependent activation of Rho kinase possibly through the phosphorylation of Rho itself[31].

Besides decreases in [Ca^2+^]_i_ and calcium sensitivity of the contractile apparatus, regulation of actin cytoskeletal dynamics have been implicated during relaxation or inhibition of contraction mediated by vasodilators [32,33,34,35,36], mainly through changes in the phosphorylation of proteins such as HSP20, cofilin, VASP and paxillin that regulate actin polymerization[37]. HSPB6 is phosphorylated by either PKA or PKG on serine 16 leading to relaxation of vascular as well as airway smooth muscle [38] by modulating actin cytoskeletal changes [37,39,40]. Phosphorylated HSPB6 has been shown to bind to the adaptor protein 14-3-3 leading to the displacement and dephosphorylation of cofilin which then acts as an actin depolymerization protein[41,42]. A phosphopeptide mimetic of HSPB6 containing the phosphorylation site, also prevents the association of 14-3-3 with cofilin allowing for disassembly of F-actin[37,43,44]. The HSPB6 protein contains a Troponin I like at amino acids 110–123 that is proposed to directly inhibit myosin binding to actin and induce relaxation [45,46]. Phosphorylation of VASP affects regulation of actin polymerization and decreases the affinity of VASP for actin by 40 fold [47]. VASP in its dephosphorylated state is involved in actin elongation, and once VASP becomes phosphorylated it loses its affinity for actin[48]. Thus sustained reduction of force mediated by cyclic nucleotides induced by vasodilators may involve multiple mechanisms that may still need to be clarified by further research.

In the operating room, papaverine is added to preservation solutions to maintain vasodilation and prevent spasm of vein grafts prior to implantation. Papaverine improves the endothelial cell viability and dilates the conduit [9]. Vasodilation is also accomplished by manual pressure distension with a hand-held syringe. Manual distension can be harmful to the graft, denuding the fragile endothelial monolayer and inducing deleterious biochemical changes[4]. The concept of “chemical distension,” in which the graft is exclusively pharmacologically distended, obviates the injury caused by manual distension. The objective of this study is to investigate the molecular mechanisms of papaverine-induced prevention of vasospasm of HSV, using alpha agonist norepinephrine as an inducer of spasm. We hypothesized that papaverine will prevent vasospasm by regulating calcium mediated myosin crossbridge phosphorylation and actin cytoskeletal dynamics. Our results show that papaverine inhibits norepinephrine-induced force and calcium flux, and regulates phosphorylation of myosin light chain and actin-associated proteins, and cortical actin polymerization. Treatment with papaverine also reduces intimal thickening in HSV in organ culture.

## Materials and Methods

### Materials

Pre-cast polyacryl amide gels, Sodium dodecyl sulfate (SDS), Tris-glycine-SDS buffer (TGS), Tris-glycine (TG) and prestained Precision Blue Protein Standards were obtained from Bio-Rad (Hercules, CA). Urea and 3-[(3-cholamidopropyl)dimethylammonio]-1-propanesulfonate (CHAPS) were obtained from Research Organics Inc. (Cleveland, OH). F/G Actin assay kit was obtained from Cytoskeleton Inc., (Denver, CO). Fura 2-AM and Pluronic F-127 were obtained from Invitrogen (Carlsbad, CA). All other chemicals were obtained from Sigma Chemical Co. (St. Louis, MO) unless specified otherwise.

### HSV procurement and physiological measurement of smooth muscle functional viability

Remnants of de-identified HSV samples were collected from patients undergoing coronary artery bypass grafting (CABG) after obtaining approval of the Institutional Review Boards of the Vanderbilt University Medical Center and the VA Tennessee Valley Healthcare System, Nashville, TN. Consent was not required as the tissue was remnant, de-identified tissue that was to be discarded; this was approved by the Vanderbilt and VA IRB. The HSV were harvested by open or minimally invasive endoscopic technique according to surgeon discretion and were stored in heparinized (10 Units/mL) PlasmaLyte (140 mEq sodium, 5 mEq potassium, 3 mEq magnesium, 98 mEq chloride, 27 mEq acetate, and 23 mEq gluconate, [Baxter Healthcare Corporation Deerfield, IL]) solution in the operating room. Only vein segments that were without damage or branches were used for analysis after careful dissection free of fat and connective tissue. One-millimeter rings from the tissue were cut, weighed and measured lengthwise using calipers. To focus only on the smooth muscle-derived changes during inhibition of force, endothelium was denuded by gently rolling the luminal surface of each ring at the tip of a fine forceps. Rings were suspended in an organ bath containing a bicarbonate buffer (120 mM NaCl, 4.7 mM KCl, 1.0 mM MgSO_4_, 1.0 mM NaH_2_PO_4_, 10 mM glucose, 1.5 mM CaCl_2_, and 25 mM Na_2_HCO_3_, pH 7.4), equilibrated with 95% oxygen and 5% carbon dioxide at 37°C. Each ring was progressively stretched to its optimal resting tension (approximately 1 g) that would produce a maximal response to contractile agonists as determined previously, then maintained at the resting tension and equilibrated for a minimum of 2 hours [49]. Force measurements were obtained using a Radnoti Glass Technology (Monrovia, CA) force transducer (159901A) interfaced with a Powerlab data acquisition system and Chart software (ADInstruments, Colorado Springs, CO). The rings were contracted first with 110 mM potassium chloride solution (KCl; with equimolar replacement of NaCl in bicarbonate buffer) to determine functional viability of the smooth muscle. Any tissue failing to contract with KCl was considered non-viable and was not used in further experiments. Viable tissues were allowed to equilibrate in the bicarbonate solution for 30 minutes and were then challenged with contractile alpha agonist norepinephrine (10^−7–^10^−5^ M) and relaxed with papaverine (10^−6^–10^-3^M). The concentration of papaverine needed to completely block norepinephrine-induced vein contraction (10^−3^ M) was determined, and used for subsequent experiments.

HSV rings were pretreated with 10^−3^ M papaverine for 10 min and then challenged with norepinephrine (5x10^-6^M-10^-5^M); the force generated was recorded. To determine the role of phosphorylation of proteins during inhibition of force, physiologic experiments were conducted and the tissues were snap frozen under tension using forceps precooled in liquid nitrogen at 5 min, and then pulverized. These pulverized tissues were stored at -80°C for analysis using urea glycerol gel, SDS polyacrylamide gel electrophoresis (PAGE) or isoelectric focusing and western blotting. For the actin assay to determine the level of F-actin compared to G-actin, the tissues were used immediately after treatment without freezing.

Contractile response was defined as stress ([10^5^ Newtons (N)/m^2^] = force (g) x 0.0987 / area, where area is equal to the wet weight [(mg) / length (mm at maximal length)] divided by 1.055),[50] which was calculated using the force (g) generated by the tissue. Percent relaxation was measured as the change in stress compared to the maximal tension induced by norepinephrine as described previously[49]. We have previously demonstrated that the production of force of less than 0.02510^5^ N/m^2^ in response to KCl correlates with diminished cellular viability as measured by the 3-(4,5-Dimethylthiazol-2-yl)-2,5-diphenyltetrazolium bromide (MTT) live/dead assay[49].

### Duration of Action of Papaverine

To determine the duration of action of papaverine, the HSV rings were equilibrated in the muscle bath as described earlier and were contracted with 5μM norepinephrine (control contraction). After maximum norepinephrine-induced contraction was reached, the rings were washed for ~1 hour. Rings were kept as untreated control or treated in duplicate, with 0.01, 0.10 or 1 mM papaverine for ten minutes before challenging with 5 μM norepinephrine (time zero contraction). After maximum contraction was reached, the rings were washed for thirty minutes, via a single buffer exchange every five minutes. The rings were subsequently re-challenged with 5 μM norepinephrine at one, two and four hours after the initial treatment. All contractions were expressed as percent of maximal (control) norepinephrine-induced contraction.

### Cytosolic Ca^2+^measurements

Cytosolic Ca^2+^ measurements were performed as described previously [51]^,^[37]. Briefly, rings of HSV were suspended on hooks in a Fluoroplex (Tissue Bath Fluorometry System, IonOptix LLC, Milton, MA), which enables fluorescence ion recording in parallel with force measurement. Force measurements were obtained with a Radnoti force transducer (Radnoti Glass Technology Inc., Monrovia, CA) interfaced with Power Lab from AD Instruments (Colorado Springs, CO). Rings were loaded at room temperature with 10 μM Fura-2 AM ester and 0.01% Pluronic F-127 in the bicarbonate buffer for 4 hrs at room temperature. After loading, rings were washed every 10 min with 37°C bicarbonate buffer for 1 hr. Calcium flux was measured with optical fibers that were interfaced with Power Lab. Fluorescence was measured at both 380 and 340 nm of wavelength, simultaneously. The ratio of the emission of the two wavelengths was used to determine intracellular changes in calcium concentration. Baseline ratio was set at 1.0 and changes in this ratio in response to stimuli were measured. Baseline calcium fluorescence was measured and the background was set to zero as an output of 1 volt. To determine the calcium response during inhibition of contraction, rings were either treated with papaverine (10^−3^ M) for 10 min, followed by norepinephrine (5 μM), or norepinephrine alone. Force and calcium fluorescence were measured continuously for 15 min after the addition of norepinephrine.

### Immunoblotting

Proteins from frozen muscle rings were extracted in UDC buffer (8 M urea, 10 mM dithiothreitol (DTT), 4% CHAPS containing protease inhibitors, and Phosphatase inhibitor I and II (Sigma, St. Louis, MO). The mixtures were vortexed at room temperature overnight, and then centrifuged at 14,000 rpm for 15 min at 4°C. Soluble protein concentrations were determined using the Coomassie Plus protein assay (Thermo Scientific, Rockfort, IL). Equal amounts (20–50 μg) of proteins were placed in a Laemmli sample buffer (Bio-Rad laboratories, Inc. Hercules, CA), heated for 5 min at 100°C and separated on SDS polyacrylamide gels. Proteins from the gels were transferred onto nitrocellulose membranes (Li-COR Biosciences, Lincoln, NE) and blocked prior to incubation overnight at 4°C with the following primary antibodies: anti-HSPB6 (1:3,000 dilution, Advanced Immunochemical Inc., Long Beach, CA); anti-VASP (1:2000, ECM Biosciences, Versailles, KY); anti-MYPT1 (1:500, Santa Cruz); anti-pMYPT1 (thr 696) (1:500, Cell Signaling); anti-MLC20 (1:7000, gift from Dr. James Stull, University of Texas, Galveston TX). Membranes were washed three times with TBS containing Tween 20 (0.1%) (TBS-T), and incubated with appropriate infrared-labeled secondary antibodies (Li-Cor, Lincoln, NE) for 1 h. at room temperature. The membranes were again washed with TBS-T, and protein-antibody complexes were visualized and quantified using the Odyssey direct infrared fluorescence imaging system (Li-COR). Phosphorylation was calculated as a ratio of the phosphorylated protein to total protein and was then normalized to the unstimulated control with the control value set as 1.0.

### Determination of myosin light chain phosphorylation

Rings of HSV were equilibrated in the muscle bath as described above and treated with norepinephrine (5X10^-6^M) for 5 min or pre-treated with papaverine (10^−3^ M) for 10 min, followed by norepinephrine for 5 min, and snap frozen as described above. Myosin light chain phosphorylation was determined using a modification of an established method described earlier [52,53]. The frozen tissue was pulverized, placed in a frozen slurry of precipitating solution consisting of 90% acetone, 10% trichloroacetic acid, and 10 mM DTT, and then allowed to melt to room temperature. The precipitating solution was removed, and the tissues were washed three times with 90% acetone and 10 mM DTT. The samples were dried, and the pellets were suspended in UDC buffer as described above and vortexed to solubilize the proteins. Ten micrograms of protein were diluted with 10 μl of urea sample buffer (6.7 M urea, 18 mM Tris, 20 mM glycine, 9 mM DTT, 4.6% saturated sucrose, and .004% bromophenol blue) and separated on glycerol-urea mini gels (40% glycerol, 10% acrylamide, 0.5% bisacrylamide, 20 mM Tris, and 22 mM glycine). Proteins were transferred onto nitrocellulose membranes in a buffer containing 10 mM Na_2_HPO_4_ pH 7.6 at 25 V for 1 hr at 20°C. The blot was probed with anti myosin light chain (MLC20) antibodies and processed as described above. The phosphorylated and non-phosphorylated MLC20 bands were quantitated by densitometric analysis. The relative amount of the phosphorylated forms of MLC20 over the total amount of MLC20 was calculated.

### Actin Assay

The amount of F-actin versus G-actin was measured using the G-actin/F-actin *In Vivo* Assay kit (Cytoskeleton, Denver, CO), per manufacturer’s protocol as described earlier[37]. Briefly, treated HSV samples were homogenized in 0.25 ml of lysis buffer (50 mM PIPES pH 6.9, 50 mM NaCl, 5 mM MgCl_2_ 5 mM EGTA, 5% (v/v) Glycerol, 0.1% Nonidet P40, 0.1% Triton X-100, 0.1% Tween 20, 0.1% 2-mercapto-ethanol, 0.001% Antifoam C, 4 μM Tosyl arginine methyl ester, 15 μM Leupeptin, 10 μM Pepstatin A, 10 mM Benzamidine, 1 mM ATP warmed to 37°C) for 1 min with a mortar and pestle that fit into the 1.5 ml microfuge tube. The lysate (100μL) was centrifuged at 2000 rpm for 5 min at 37°C to pellet unbroken cells. The supernatants were centrifuged at 100,000 g for 1 hr at 37°C. Supernatants (contains the G-actin) were transferred to pre-cooled tubes and placed on ice. The pellets (contain F-actin) were resuspended in 100μL of ice-cold 10 μM cytochalasin D in deionized water, and F- actin was depolymerized by incubating for 1 hr on ice with mixing every 15 min. Equal volume of supernatants and pellets along with actin standards (50-100ng) were separated on 12% SDS-polyacrylamide gels and transferred to nitrocellulose membrane in 1X TG buffer at 100 volts for 1 hr. The membrane was probed with anti actin antibody(1:1000 dilution cytoskeleton) and the amount of actin in each fraction was quantified comparing to actin standards loaded on the same gel.

### Isoelectric focusing

Phosphorylation of HSPB6 in response to vasodilators was examined by isoelectric focusing, which separates the phosphorylated and non-phosphorylated forms of HSPB6 and detected by western blotting. 30 μg of extracted proteins from the treated HSV samples were separated on one-dimensional isoelectric focusing gel (8.3X7.3 cm) with 5% ampholines (4 parts pI 4–7 and 1 part pI 3–10, GE Healthcare Bio-Sciences) using 20 mM sodium hydroxide as a cathode buffer and 10 mM phosphoric acid as an anode buffer. Proteins were focused for 100 V for 1 hr, 250 V for 1 hr and 500 V for 30 min and transferred to nitrocellulose membrane at 25 V in 0.7% acetic acid with the direction of the gel sandwich reversed (acetic acid give proteins a positive charge) for 1 hr at room temperature. The blot was probed with anti-HSPB6 antibodies and the phosphorylated and non-phosphorylated forms of HSPB6 were quantitated by densitometry. The ratio of phospho-HSPB6 to total HSPB6 was calculated and normalized to the control untreated tissue.

### Human saphenous vein organ culture and morphometric analyses

Effect of papaverine on intimal hyperplasia formation *ex vivo* was determined by vein organ culture. Two rings of HSV from each patient were placed in 10% neutral buffered formalin to measure the basal intimal thickness. To measure intima development *in vitro*, additional rings were placed in 8-well chamber slides, pretreated with or without papaverine (10^−3^) for 2 hours, and maintained in RPMI 1640 medium supplemented with 30% FBS (Gibco, Carlsbad, CA), 1% L-glutamine, and 1% penicillin/streptomycin for 14 days at 37˚C in an atmosphere of 5% CO_2_ in air. The culture medium was replaced every 2–3 days. After 14 days rings were fixed in 10% formalin, and sent to the Pathology Histochemistry Core at Vanderbilt University for histological preparation. The rings were embedded in paraffin, sectioned (5 μm) and multiple sections were stained using Movat to allow the visualization of the internal elastic lamina. Measurements of intimal thickness were made on transverse sections of each vessel using a Zeiss Axiovert 200M microscope (Carl Zeiss, Thornwood, N.Y., USA) with a computerized image analysis system (Zeiss software and Adobe Photoshop). Intima was defined as tissue on the luminal side of the internal elastic lamina. For each ring, four measurements were made in each image, one from each quadrant, for 3 sections for a total of 12 measurements. The mean intimal thickness was the average of 24 measurements on 6 histological sections from 2 rings from a single human saphenous vein. The images at 5X maginification were also uploaded into MATLab for area measurements. Using the imagesc tool with roipoly, the user is able to select a region of interest where the area may be measured. The area of the intima for each saphenous vein segment was taken and plotted.

### Statistical analysis

Values are reported as mean ± standard error of the mean (SEM). Statistical analysis was performed by unpaired Student’s *t* test or one-way *ANOVA*, followed by Tukey’s post-test (GraphPad Software, Inc. San Diego, CA). The criterion for significance was *P* < 0.05.

## Results

### The effect of papaverine on inhibition of contraction

**Fig 1:**
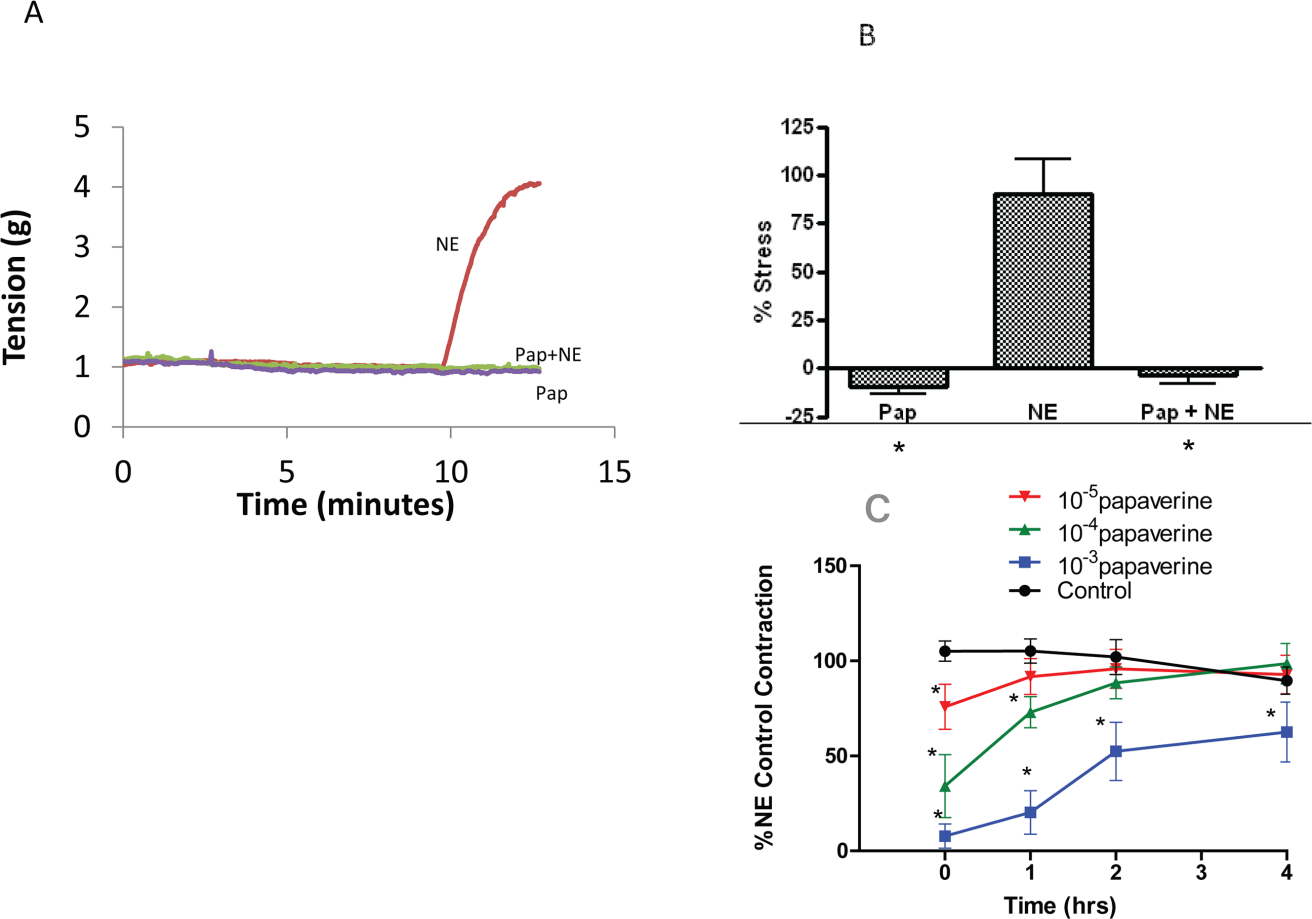
Preincubation with papaverine inhibits norepinephrine (NE) induced contractions in human saphenous vein. Saphenous vein rings were suspended in a muscle bath and equilibrated in Kreb’s bicarbonate buffer for 2 h. Panel A: Representative force tracings of rings treated with 0.5 μM NE, 1mM papaverine (Pap), or 1 mM papaverine for 10 min followed by 0.5 μM NE. Panel B: Cumulative data obtained when the force was converted to stress and decrease in stress was converted to a percentage of the maximal initial KCl contraction which was set as 100%. Means ± SE, n = 6 P < 0.05. * Significant compared to NE. Panel C:

To simulate the prevention of vasospasm during HSV preparation, papaverine was used to pretreat the tissue followed by treatment with norepinephrine. Initial experiments were conducted using various doses of norepinephrine to contract HSV, and the dose of norepinephrine (5x10^-6^M-10^-5^M) which produced greater than 60% of maximal potassium-induced contraction was selected for subsequent experiments. Various doses of papaverine (0.1–1 mM) were used to block norepinephrine-induced contraction and a dose of 1 mM that completely blocked norepinephrine-induced contraction was chosen for this study. Treatment of HSV with norepinephrine alone generated force (89 ± 18% of KCl stress n = 6 Fig 1B). Pre-treatment of HSV with 1 mM papaverine blocked norepinephrine-induced force generation (-3 ± 5% p<0.05, n = 6 Fig 1B). Papaverine-induced inhibition of force was reversible as washing the rings repeatedly allowed the HSV to contract to (5x10^-6^M) norepinephrine in a time-dependent manner (Fig 1C), and demonstrated that the doses of norepinephrine and papaverine used in this study did not affect the viability of the tissue. The dose of papaverine used for this study is similar to the dose used clinically, 1mg/ml, and this dose has a long duration of action (4 hrs, Fig 1C).

### The effect of papaverine on Ca^2+^ transients

**Fig 2:**
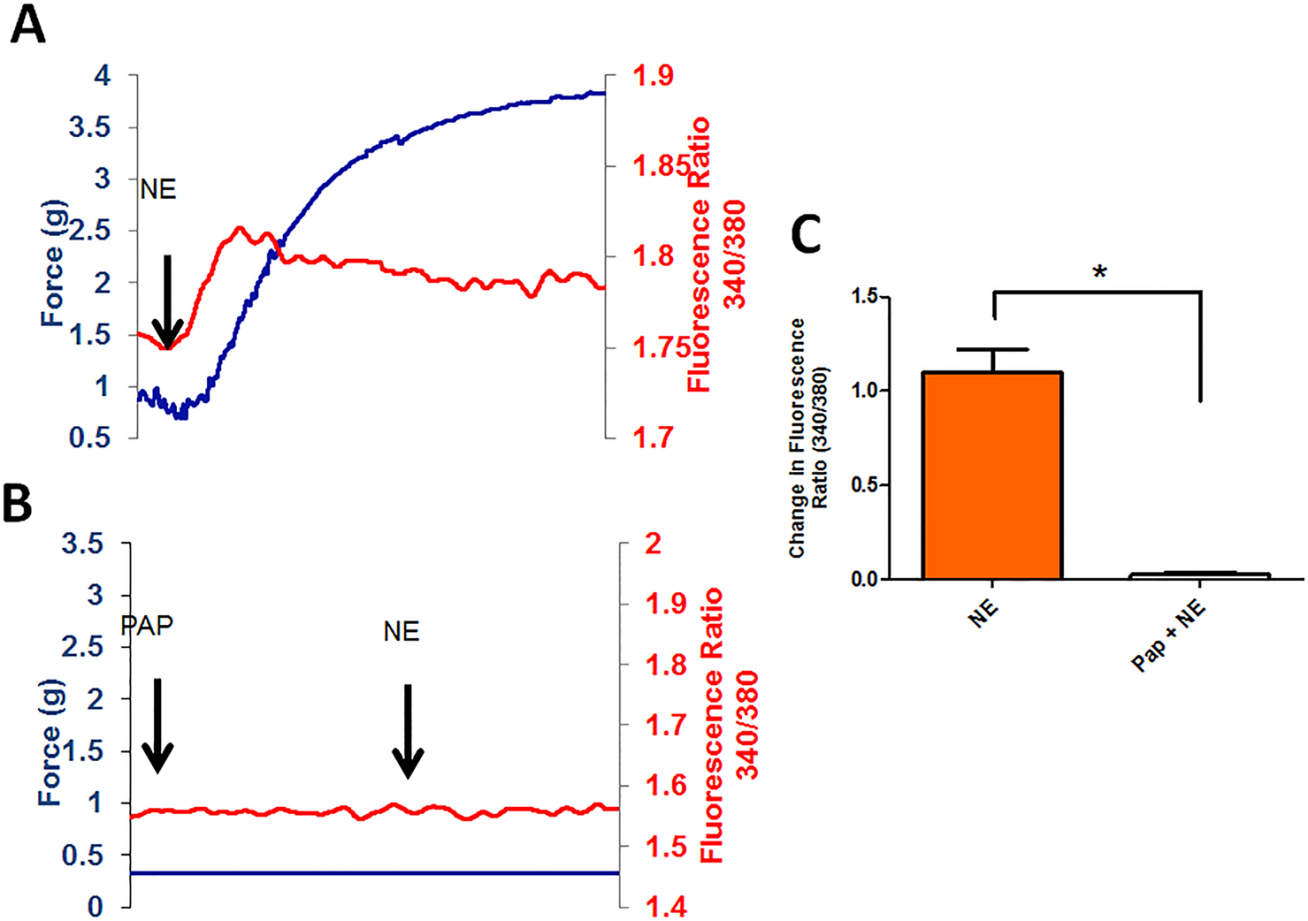
Pre incubation with papaverine, blocks NE induced isometric force generation (blue, Panel A and B) and intracellular Ca^2+^ release (red, Panel A and B) in HSV. HSV was suspended on a Fluroplex apparatus and loaded with Fura-2AM for 4 hrs, then treated with either norepinephrine alone or papaverine (1mM) pretreatment followed by norepinephrine. Tissue treated with papaverine had significantly lower changes in fluorescence ratio (N = 3, * Significant compared to NE, p<0.05 Panel C).

Papaverine acts as a phosphodiesterase inhibitor leading to vasodilation of vascular smooth muscle. Understanding Ca^2+^ transients in response to inhibition of force allows a better understanding of the mechanism of inhibition of contraction. Norepinephrine treatment led to an increase in [Ca^2+^]_i_ followed by force generation (1.10 ± 0.12 A.U., n = 3, Fig 2). Pre-treatment with papaverine significantly inhibited [Ca^2+^]_i_ and force generation in response to norepinephrine (0.01 ± 0.01 A.U., p = 0.013, n = 3).

### The effect of papaverine on myosin light chain phosphorylation

**Fig 3:**
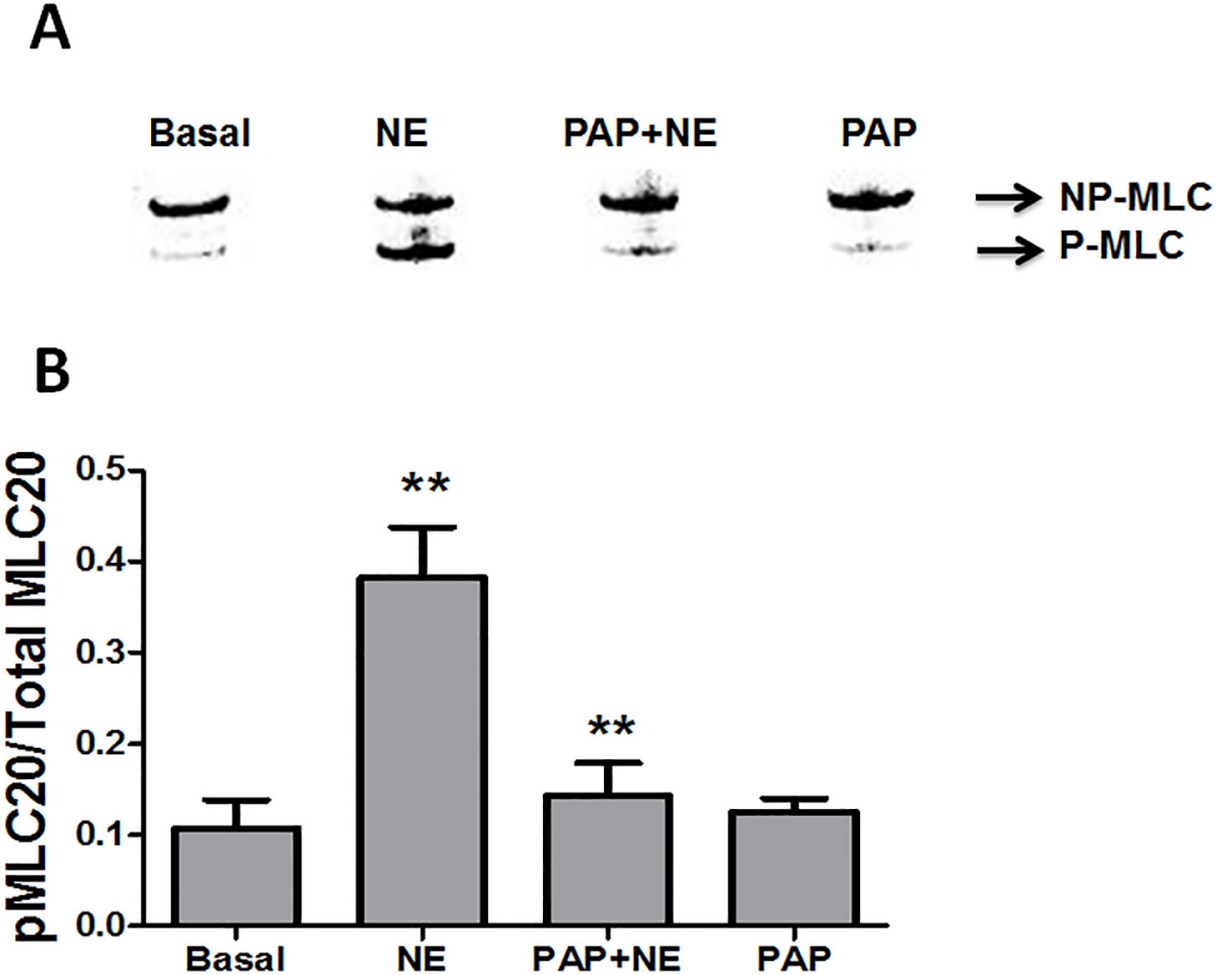
Preincubation with papaverine leads to decreases in phosphorylation of the myosin light chain in HSV. HSV rings were suspended in a muscle bath and equilibrated in Kreb’s bicarbonate buffer for 2 h. Rings were untreated (Basal) or treated with 1 μM norepinephrine (NE) for 5 minutes, 1 mM papaverine (Pap) for 10 min followed by 1 μM norepinephrine for 5 min or 1 mM papaverine (Pap) for 10 min,. Tissues were snap frozen and ratios of phosphorylated myosin light chain to non-phosphorylated myosin light chain was calculated. Representative blots are shown in Panel A with the cumulative bar graphs shown in Panel B. N = 4, * Significant compared to p<0.05.

Papaverine treatment decreased [Ca^2+^]_i_, and since myosin light chain (MLC20) phosphorylation correlates with changes in [Ca^2+^]_I_, papaverine pretreatment should prevent increases in MLC20 phosphorylation. Norepinephrine treatment significantly increased the phosphorylation of MLC20 (0.382 ± 0.06 Mol Pi/Mol MLC20) when compared to untreated basal (0.11 ± 0.03 Mol Pi/MolMLC20) (n = 4, p = 0.01). Pretreatment with papaverine followed by norepinephrine treatment significantly inhibited the phosphorylation of MLC20 (0.14 ± 0.04 Mol Pi/Mol MLC20) when compared to norepinephrine alone (0.382 ± 0.06 Mol Pi/MolMLC20, n = 3, p = 0.02, Fig 3).

### Papaverine treatment decreases filamentous actin levels

**Fig 4:**
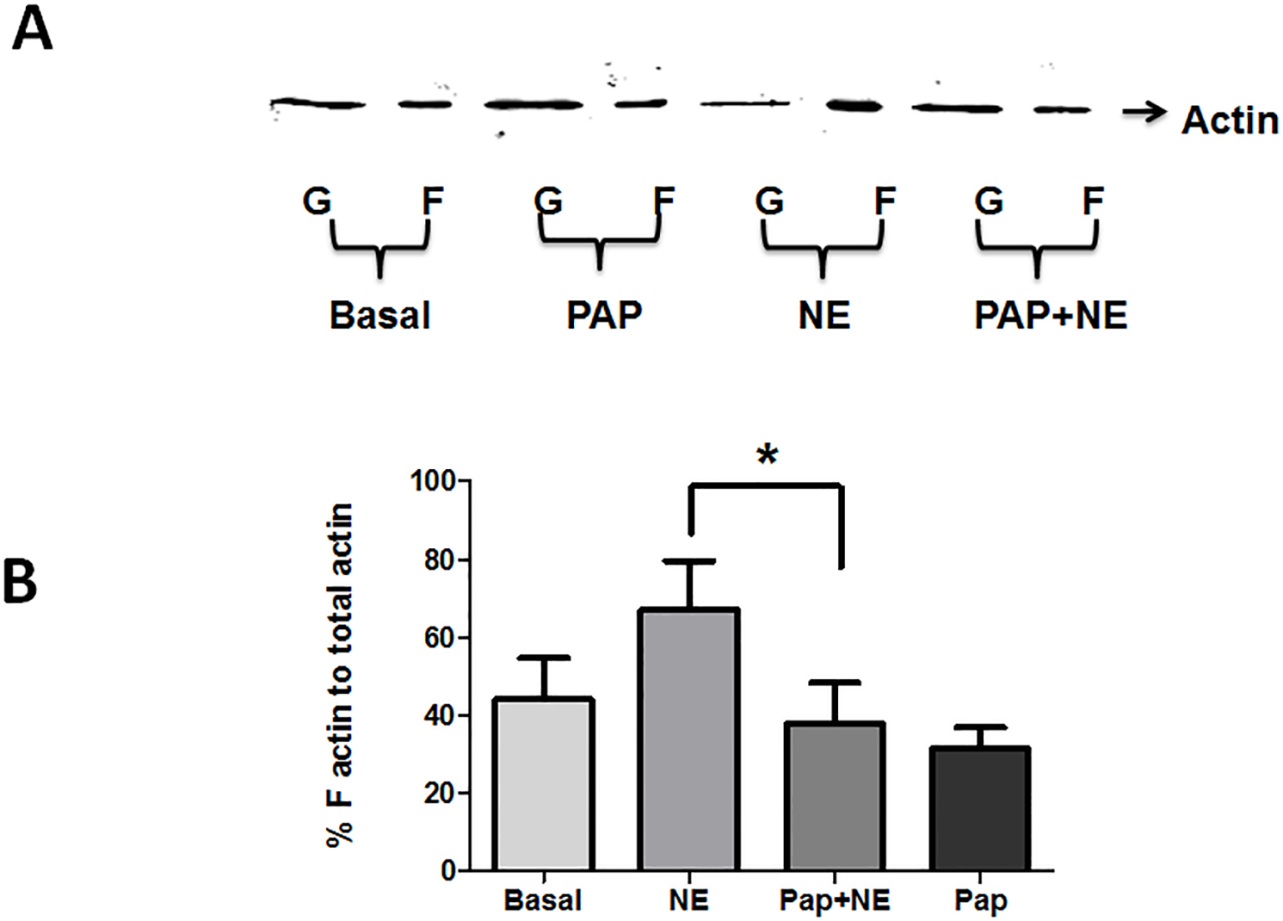
Preincubation with papaverine leads to decreases in F-actin in HSV. HSV rings were suspended in a muscle bath and equilibrated in Kreb’s bicarbonate buffer for 2 h. Rings were untreated (Basal) or treated with 1 mM papaverine (Pap) for 10 min, 1 μM norepinephrine (NE) for 5 minutes, or 1 mM papaverine (Pap) for 10 min followed by 1 μM norepinephrine for 5 min. Tissues were snap frozen and F- and G-actin were separated by centrifugation and F- actin was quantitated by western blotting and densitometry. % F-actin to total actin (F+G) was calculated. Representative western blots are shown in Panel A with the cumulative bar graphs shown in Panel B. N = 4, * Significant compared to NE, p<0.05.

Several investigators have demonstrated that actin is polymerized during contraction of smooth muscle, and agents that inhibit actin polymerization result in inhibition of contraction (reviewed in [54]). We determined whether papaverine treatment had an effect on actin polymerization. HSV was treated with either buffer alone (basal), norepinephrine (1x10^-6^M, 5 min), pretreated with papaverine (1 mM, 10 min) followed by norepinephrine (1x10^-6^M, 5 min), or papaverine alone (1 mM, 10 min), and changes in actin polymerization was analyzed by measuring F and G-actin level using a F/G actin assay. Treatment with norepinephrine led to increases in F-actin by 13% (44 ± 10% to 67 ± 12% for basal and norepinephrine, respectively, p<0.05, n = 5), while treatment with papaverine reduced F-actin by 12% (44 ± 10% to 32 ± 5% for basal and papaverine, respectively). Pretreatment with papaverine for 10 min before norepinephrine stimulation reduced the F- actin by 29% (67 ± 12% to 38 ± 11% for basal and papaverine + norepinephrine, respectively *p* = 0.004, n = 5, Fig 4).

### The effect of papaverine on the phosphorylation of actin regulating proteins, HSPB6 and VASP

**Fig 5:**
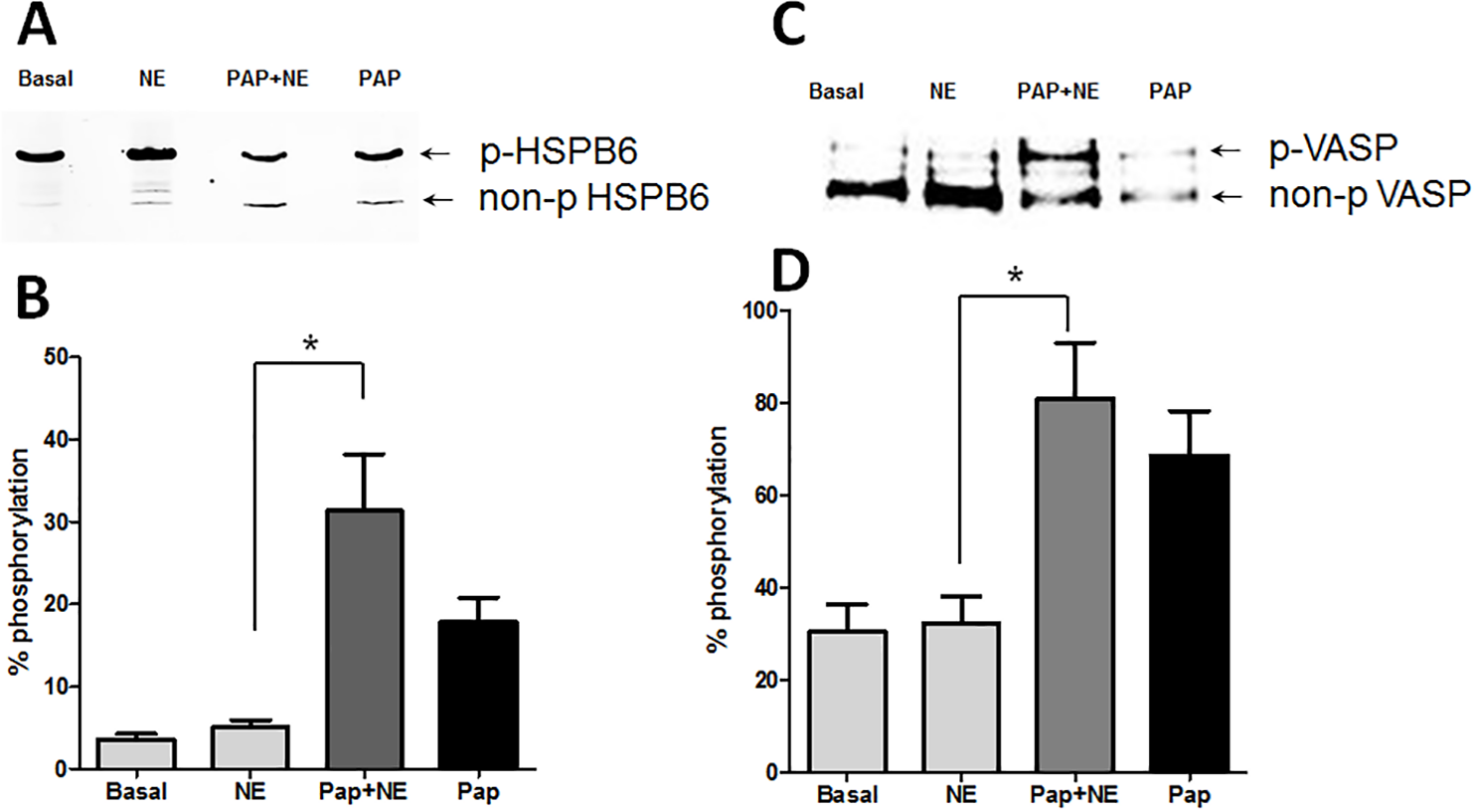
Preincubation with papaverine leads to increases in phosphorylation of the HSPB6 and VASP in HSV. HSV rings were suspended in a muscle bath and equilibrated in Kreb’s bicarbonate buffer for 2 h. Rings were untreated (Basal), treated with 1 μM norepinephrine (NE) for 5 minutes, 1 mM papaverine (Pap) for 10 min followed by 1 μM norepinephrine for 5 min or 1 mM papaverine (Pap) for 10 min. Tissues were snap frozen and ratios of p-HSPB6/total HSPB6 and p-VASP/total VASP to were calculated. Representative western blot for HSPB6 are shown in Panel A with the cumulative bar graph shown in Panel B. Representative blot of VASP are shown in Panel C with the cumulative bar graph in Panel D. * Significant compared to NE, N = 4, p<0.05.

Treatment of smooth muscle with vasodilators or phosphodiesterase inhibitors such as papaverine leads to increases in the phosphorylation of HSPB6 and induces relaxation [55]. To determine the role of HSPB6 phosphorylation in the regulation of actin polymerization during force inhibition, HSV was treated with either basal conditions, norepinephrine, papaverine followed by norepinephrine, or papaverine alone. HSPB6 phosphorylation was examined by isoelectric focusing and western blot analysis. Papaverine led to increases in the phosphorylation of HSPB6, [5.03 ± 0.91 and 31.30 ± 6.96 phospho-HSPB6/total HSPB6 for norepinephrine and papaverine + norepinephrine, respectively (*p* < 0.05, n = 4)] in HSV. HSPB6 remained phosphorylated even after norepinephrine was added to induce contraction (Fig 5A).

Next, the effects of papaverine on VASP phosphorylation, which have been demonstrated to regulate actin polymerization, was determined. VASP was phosphorylated in response to papaverine, but not norepinephrine treatment, and the phosphorylation was not reversed by norepinephrine treatment after papaverine (32.21 ± 5.74 and 81.03 ± 12.12 p-VASP/VASP for norepinephrine and papaverine + norepinephrine, respectively *p* < 0.05, n = 4, Fig 5B).

### Papaverine treatment prevents intimal thickening in culture

**Fig 6:**
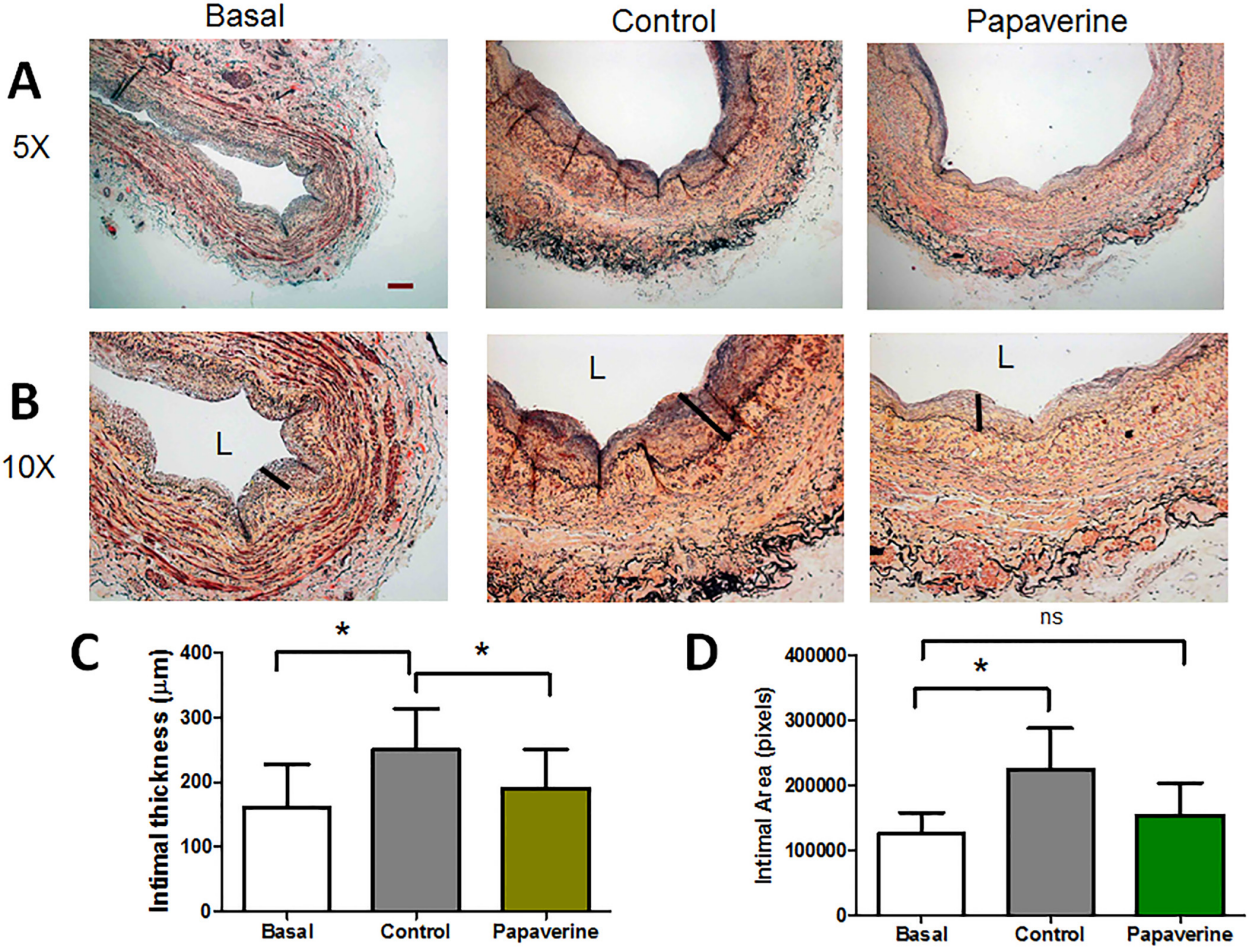
Papaverine pretreatment for 2 hrs prior to organ culture attenuated intimal thickening in HSV. HSV were fixed at day 0 for preculture (basal) conditions and then at 14 days with either control treatment or pretreatment with 1 mM papaverine for 2 hrs. Tissue was fixed after 14 days of organ culture and the intimal thickness was measured. Representative staining shown in 5 x magnification are shown in Panel A, with 10 x magnification shown in Panel B. Black line indicates the intimal thickening. The cumulative quantification of intimal thickness is displayed in Panel C. * Significant compared to control, (N = 4), p<0.05 The area of the thickness was measured using Matlab software and the cumulative data is shown in Panel D. * Significant compared to control, N = 4, p<0.05.

Intimal hyperplasia is the leading cause of vein graft failure[56,57], thus we investigated whether the treatment of vein with papaverine during grafting to prevent spasm will also have an added effect on the intimal hyperplasia formation after implantation. We chose a well characterized *in vitro* organ culture model of intimal hyperplasia formation in the vein which has been described before[4]. Basal intimal thickness of the vein segments were (160.7 μm± 67.3). Organ culture led to a significant increase in intimal thickness (250.0 μm± 63.1, *p*<0.05, n = 3, Fig 6). Papaverine treatment (1 mM, 2 hrs) significantly inhibited the increase in intimal thickness after organ culture (190.7 μm± 59.9). Area of the intima at 5X magnification of the vein segments were 125978 pixels± 32544. Organ culture led to a significant increase in intimal area (225203 pixels ± 62667, *p*<0.05, n = 3, Fig 6). Papaverine treatment (1 mM, 2 hrs) significantly inhibited the increase in intimal area after organ culture (153711 pixels ± 49773).

### The effect of papaverine treatment on MYPT1 phosphorylation

**Fig 7:**
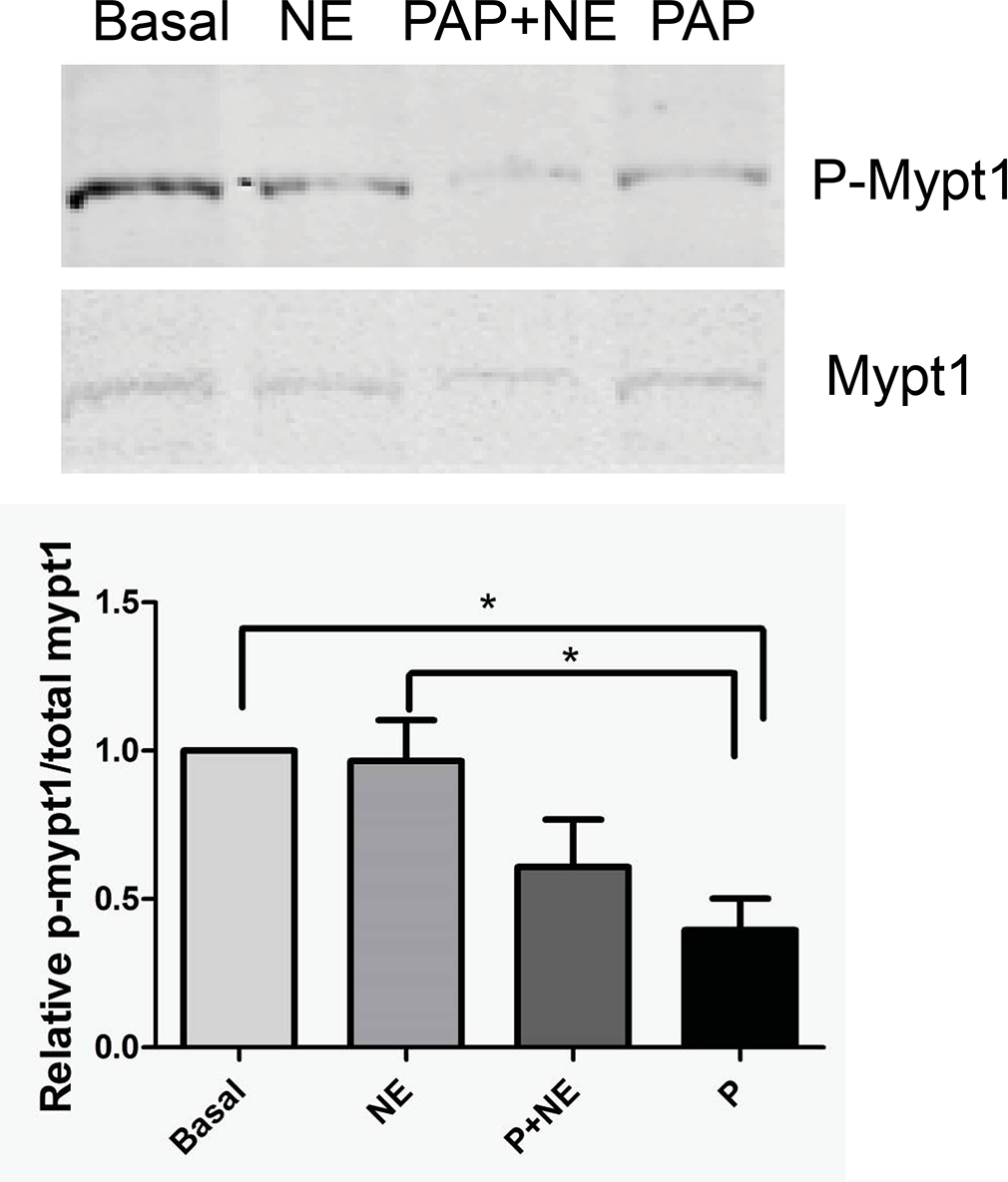
Preincubation with papaverine leads to decreases in phosphorylation of the mypt1 in HSV. HSV rings were suspended in a muscle bath and equilibrated in Kreb’s bicarbonate buffer for 2 h. Rings were untreated (Basal), treated with 1 μM norepinephrine (NE) for 5 minutes, 1 mM papaverine (Pap) for 10 min followed by 1 μM norepinephrine for 5 min or 1 mM papaverine (Pap) for 10 min. Tissues were snap frozen and protein lysates were separated on 4–20% criterion gels and transferred to nitrocellulose membrane. Mypt1 and phospho mypt1 were identified by western blotting using antibodies against mypt1(Santa Cruz, 1:500) and phospho mypt1(thr 696) antibodies (cell signal, 1:500), respectively, and the ratio of p-mypt1/total mypt1 was calculated. Representative western blot for mypt1 are shown in Panel A with the cumulative bar graph shown in Panel B. * Significant, N = 4, p<0.05.

To determine the role of papaverine in MYPT1 phosphorylation, HSV was treated with either basal conditions, norepinephrine, papaverine followed by norepinephrine, or papaverine alone. MYPT1 phosphorylation was examined by western blot analysis. Papaverine led to decreases in the phosphorylation of MYPT1, [0.97 ± 0.14 and 0.40 ± 0.11 phospho-MYPT1/total MYPT1 for norepinephrine and papaverine, respectively (*p* < 0.05, n = 4)] in HSV. MYPT1 phosphorylation had had a non-significant decrease in tissue pretreated with papaverine and then treated with norepinephrine (Fig 7).

## Discussion

Papaverine is a long acting phosphodiesterase inhibitor which leads to accumulation of cAMP and cGMP, thus making it a useful pharmacologic agent for preventing vasospasm in HSV. We have demonstrated that papaverine can regulate the intracellular calcium mediated/myosin light chain phosphorylation pathway as well as actin regulatory protein mediated actin depolymerization pathway, both of which have been implicated in relaxation of smooth muscle tissue [12,40],[35].

In this study, a physiologic system was developed in which vasospasm of HSV was induced by treatment with norepinephrine and the mechanism of papaverine-induced prevention of vasospasm was examined. Treatment of HSV with papaverine resulted in complete suppression of force generation in the presence of a contractile agonist, norepinephrine (Fig 1). This physiologic model allowed us to examine force development and [Ca^2+^]_i_ concurrently using the FluoroPlex Tissue Bath Fluorometry System and to decipher the role of actin cytoskeletal dynamics (thin filaments) during inhibition of contraction or force suppression. The decreases in force and [Ca^2+^]_i_ were correlated with decreased myosin light chain phosphorylation levels which demonstrates that papaverine inhibits myosin cross bridge phosphorylation (Fig 3).

More recently, several investigations have suggested the regulation of actin and actin-associated proteins in smooth muscle contraction and we examined the effect of papaverine on actin polymerization and the phosphorylation of actin associated proteins[54]. We have demonstrated earlier that increasing cAMP levels with forskolin leads to depolymerization of actin and phosphorylation of actin regulatory proteins such as HSPB6 and VASP [35]. Papaverine treatment also led to increases in the phosphorylation of HSPB6 and VASP (Fig 5). Papaverine also increased G-actin levels indicating depolymerization of actin (Fig 4) similar to the results we obtained with forskolin in pig coronary artery where HSPB6 phosphorylation induced actin depolymerization and relaxation [35]. Ba *et al* have reported that HSPB6 phosphorylation causes relaxation through depolymerization of F-actin as well as inhibition of myosin binding to actin [58]. We have reported earlier that HSPB6 phosphorylation mediates relaxation through displacement of cofilin from 14-3-3 leading to dephosphorylation and activation as a actin depolymerizing factor [59]. However, studies have shown that cofilin only weakly interacts with 14-3-3 and therefore cannot directly compete with phosphorylated HSPB6 for binding to 14-3-3 [60]. These authors hypothesize that phosphorylated HSPB6 affect interaction of 14-3-3 with protein phosphatases and/or kinases involved in dephosphorylation or phosphorylation of cofilin thereby regulating reorganization of actin cytoskeleton[60]. Phosphorylation of VASP affects regulation of actin polymerization and decreases the affinity of VASP for actin by 40 fold [47]Thus, the increases in phosphorylation of VASP associated with papaverine treatment may prevent F-actin polymerization when HSV is treated with norepinephrine. Collectively, papaverine mediated phosphorylation of HSPB6 and VASP induce actin cytoskeletal changes leading to sustained inhibition of contraction in HSV through multiple mechanisms.

To directly assess the level of actin depolymerization induced by papaverine, we measured the F-actin level. Papaverine treatment decreased basal F- actin levels and also prevented the increase in F-actin induced by norepinephrine (Fig 4). We have reported earlier that cAMP inhibits histamine induced F-actin levels in porcine coronary artery and actin (thin filament) depolymerization has been demonstrated to be associated with force inhibition[37]. Papaverine pretreatment inhibited the formation of F-actin upon the addition of norepinephrine in human saphenous vein segments.

Papaverine is a phosphodiesterase inhibitor, which is able to inhibit the hydrolysis of cAMP and cGMP in tissue. It has been shown that increases in cAMP and cGMP reduce intimal hyperplasia in an organ culture model[61,62,63,64]. Papaverine was able to attenuate generation of a deleterious neointimal formation. Moreover, its ability to regulate both the thick and thin filament dynamic confers against vasospasm *ex* vivo, to a greater degree than commonly employed anti-spasmodic graft manipulations, such as high pressure distension that may promote endothelial denudation and its sequelae[65]. The ability for papaverine to inhibit culture induced intimal hyperplasia makes it a superior alternative to distension of saphenous veins during back table preparation. Since the primary cause for graft failure in coronary artery bypass grafting is from intimal hyperplasia, the reduction of this response presents as a potentially beneficial therapeutic to increase graft patency[66].

While our findings suggest the clinically relevant mechanism for papaverine-induced prevention of vasospasm, there were several limitations. First, cAMP and cGMP levels in the tissue upon treatment of papaverine were not quantified. Next, saphenous vein spasm manifests ostensibly due to multiple vasoactive agents that were not assessed, including circulating serotonin, endothelin and other catecholamines beyond norepinephrine, allowing for the potential involvement of alternative pathways in the pathogenesis of vasospasm. We have also not addressed other mechanisms of papaverine-induced inhibition of contraction such as Rho kinase-mediated activation of myosin phosphatase. Additionally, it would be important to study to the long-term survival of these grafts as opposed to short term organ culture models where the end point is 14 days. Finally, the organ culture model used had no flow to mimic physiologic shear stresses that a conduit would normally encounter upon arterialization. Despite the limitations, our study was conducted with intact tissue, making it more germane than cell culture experiments.

### Significance

Treatment of HSV with papaverine inhibits force production by norepinephrine. This inhibition of force production was associated with inhibition of [Ca^2+^]_I_ and myosin light chain phosphorylation, suggesting thick filament mechanism of force inhibition. In addition inhibition of force production was associated with increases in the phosphorylation of the actin regulatory proteins HSPB6 and VASP and decreases in filamentous actin suggesting actin cytoskeletal mechanism of force inhibition. Thus, papaverine likely inhibits vasospasm through multiple regulatory processes. As data emerges describing the cellular harm caused by manual distention[65], papaverine may represent a preferred approach for the prevention of vasospasm in vein graft preparation.

## Abbreviations

HSV: Human Saphenous Vein
CABG: Coronary Artery Bypass Grafting
IMA: Internal Mammary Artery
SNP: Sodium Nitroprusside
PKG: Protein Kinase G
[Ca2+]I: Intracellular Calcium Concentratoin
PKA: Protein Kinase A
MLC20: Myosin Light Chain 20

## References

[1] Sogo N, Campanella C, Webb DJ, Megson IL (2000) S-nitrosothiols cause prolonged, nitric oxide-mediated relaxation in human saphenous vein and internal mammary artery: therapeutic potential in bypass surgery. British journal of pharmacology 131: 1236–1244. 11082133 10.1038/sj.bjp.0703700 PMC1572448

[2] Grondin CM, Campeau L, Thornton JC, Engle JC, Cross FS, et al (1989) Coronary artery bypass grafting with saphenous vein. Circulation 79: I24–29. 2655977

[3] Rosenfeldt FL, He GW, Buxton BF, Angus JA (1999) Pharmacology of coronary artery bypass grafts. Ann Thorac Surg 67: 878–888. 10215263 10.1016/s0003-4975(98)01299-5

[4] Li FD, Eagle S, Brophy C, Hocking KM, Osgood M, et al (2014) Pressure Control During Preparation of Saphenous Veins. JAMA surgery. 10.1001/jamasurg.2013.5067 PMC4102634 24759942

[5] Motwani JG, Topol EJ (1998) Aortocoronary saphenous vein graft disease: pathogenesis, predisposition, and prevention. Circulation 97: 916–931. 9521341 10.1161/01.cir.97.9.916

[6] Kerz T, Boor S, Beyer C, Welschehold S, Schuessler A, et al (2012) Effect of intraarterial papaverine or nimodipine on vessel diameter in patients with cerebral vasospasm after subarachnoid hemorrhage. Br J Neurosurg 26: 517–524. 10.3109/02688697.2011.650737 22303863

[7] Cairns CJ, Finfer SR, Harrington TJ, Cook R (2003) Papaverine angioplasty to treat cerebral vasospasm following traumatic subarachnoid haemorrhage. Anaesth Intensive Care 31: 87–91. 12635402 10.1177/0310057X0303100117

[8] Mindea SA, Yang BP, Bendok BR, Miller JW, Batjer HH (2006) Endovascular treatment strategies for cerebral vasospasm. Neurosurgical focus 21: E13. 10.3171/foc.2006.21.3.13 17029337

[9] Kaneda T, Shimizu K, Nakajyo S, Urakawa N (1998) The difference in the inhibitory mechanisms of papaverine on vascular and intestinal smooth muscles. European journal of pharmacology 355: 149–157. 9760029 10.1016/s0014-2999(98)00479-8

[10] Warton A, Papadimitriou JM, Goldie RG, Paterson JW (1986) An ultrastructural study of mast cells in the alveolar wall of normal and asthmatic lung. The Australian journal of experimental biology and medical science 64 (Pt 5): 435–444. 3579738 10.1038/icb.1986.46

[11] Lincoln TM, Komalavilas P, Boerth NJ, MacMillan-Crow LA, Cornwell TL (1995) cGMP signaling through cAMP- and cGMP-dependent protein kinases. Adv Pharmacol 34: 305–322. 8562442 10.1016/s1054-3589(08)61094-7

[12] Lincoln TM, Dey N, Sellak H (2001) Invited review: cGMP-dependent protein kinase signaling mechanisms in smooth muscle: from the regulation of tone to gene expression. J Appl Physiol 91: 1421–1430. 11509544 10.1152/jappl.2001.91.3.1421

[13] Cornwell TL, Lincoln TM (1989) Regulation of intracellular Ca2+ levels in cultured vascular smooth muscle cells. Reduction of Ca2+ by atriopeptin and 8-bromo-cyclic GMP is mediated by cyclic GMP-dependent protein kinase. The Journal of biological chemistry 264: 1146–1155. 2536016

[14] Schultz K, Schultz G (1977) Sodium nitroprusside and other smooth muscle-relaxants increase cyclic GMP levels in rat ductus deferens. Nature 265: 750–751. 193029 10.1038/265750a0

[15] Hidaka H, Yamaki T, Naka M, Tanaka T, Hayashi H, et al (1980) Calcium-regulated modulator protein interacting agents inhibit smooth muscle calcium-stimulated protein kinase and ATPase. Molecular pharmacology 17: 66–72. 6247638

[16] Blatz AL, Magleby KL (1984) Ion conductance and selectivity of single calcium-activated potassium channels in cultured rat muscle. The Journal of general physiology 84: 1–23. 6086805 10.1085/jgp.84.1.1 PMC2228730

[17] Kamm KE, Stull JT (1985) The function of myosin and myosin light chain kinase phosphorylation in smooth muscle. Annual review of pharmacology and toxicology 25: 593–620. 2988424 10.1146/annurev.pa.25.040185.003113

[18] Somlyo AP, Somlyo AV (1994) Signal transduction and regulation in smooth muscle. Nature 372: 231–236. 7969467 10.1038/372231a0

[19] Rasmussen H, Takuwa Y, Park S (1987) Protein kinase C in the regulation of smooth muscle contraction. FASEB journal: official publication of the Federation of American Societies for Experimental Biology 1: 177–185. 3040504

[20] van Breemen C, Saida K (1989) Cellular mechanisms regulating [Ca2+]i smooth muscle. Annual review of physiology 51: 315–329. 2653185 10.1146/annurev.ph.51.030189.001531

[21] Alexander RW, Brock TA, Gimbrone MA Jr, Rittenhouse SE (1985) Angiotensin increases inositol trisphosphate and calcium in vascular smooth muscle. Hypertension 7: 447–451. 2987120

[22] Karaki H, Ozaki H, Hori M, Mitsui-Saito M, Amano K, et al (1997) Calcium movements, distribution, and functions in smooth muscle. Pharmacological reviews 49: 157–230. 9228665

[23] Alioua A, Huggins JP, Rousseau E (1995) PKG-I alpha phosphorylates the alpha-subunit and upregulates reconstituted GKCa channels from tracheal smooth muscle. Am J Physiol 268: L1057–L1063. 7611428 10.1152/ajplung.1995.268.6.L1057

[24] Raeymaekers L, Hofmann F, Casteels R (1988) Cyclic GMP-dependent protein kinase phosphorylates phospholamban in isolated sarcoplasmic reticulum from cardiac and smooth muscle. The Biochemical journal 252: 269–273. 2844148 10.1042/bj2520269 PMC1149133

[25] Komalavilas P, Lincoln TM (1994) Phosphorylation of the inositol 1,4,5-trisphosphate receptor by cyclic GMP-dependent protein kinase. J Biol Chem 269: 8701–8707. 8132598

[26] Koga T, Yoshida Y, Cai JQ, Islam MO, Imai S (1994) Purification and characterization of 240-kDa cGMP-dependent protein kinase substrate of vascular smooth muscle. Close resemblance to inositol 1,4,5-trisphosphate receptor. J Biol Chem 269: 11640–11647. 8157697

[27] Somlyo AP, Somlyo AV (2003) Ca2+ sensitivity of smooth muscle and nonmuscle myosin II: modulated by G proteins, kinases, and myosin phosphatase. Physiol Rev 83: 1325–1358. 14506307 10.1152/physrev.00023.2003

[28] Surks HK, Mochizuki N, Kasai Y, Georgescu SP, Tang KM, et al (1999) Regulation of myosin phosphatase by a specific interaction with cGMP- dependent protein kinase Ialpha. Science 286: 1583–1587. 10567269 10.1126/science.286.5444.1583

[29] Pfeifer A, Nurnberg B, Kamm S, Uhde M, Schultz G (1995) Cyclic GMP-dependent protein kinase blocks pertussis toxin-sensitive hormone receptor signaling pathways in Chinese Hamster Ovary cells. J Biol Chem 270: 9052–9059. 7721818 10.1074/jbc.270.16.9052

[30] Lee MR, Li L, Kitazawa T (1997) Cyclic GMP causes Ca2+ desensitization in vascular smooth muscle by activating the myosin light chain phosphatase. The Journal of biological chemistry 272: 5063–5068. 9030570 10.1074/jbc.272.8.5063

[31] Sauzeau V, Le Jeune H, Cario-Toumaniantz C, Smolenski A, Lohmann SM, et al (2000) Cyclic GMP-dependent protein kinase signaling pathway inhibits RhoA-induced Ca2+ sensitization of contraction in vascular smooth muscle. The Journal of biological chemistry 275: 21722–21729. 10783386 10.1074/jbc.M000753200

[32] McDaniel NL, Chen XL, Singer HA, Murphy RA, Rembold CM (1992) Nitrovasodilators relax arterial smooth muscle by decreasing [Ca2+]i and uncoupling stress from myosin phosphorylation. Am J Physiol 263: C461–467. 1325117 10.1152/ajpcell.1992.263.2.C461

[33] Woodrum DA, Brophy CM, Wingard CJ, Beall A, Rasmussen H (1999) Phosphorylation events associated with cyclic nucleotide-dependent inhibition of smooth muscle contraction. Am J Physiol 277: H931–939. 10484413 10.1152/ajpheart.1999.277.3.H931

[34] Gerthoffer WT, Murphy RA (1983) Myosin phosphorylation and regulation of cross-bridge cycle in tracheal smooth muscle. Am J Physiol 244: C182–187. 6829745 10.1152/ajpcell.1983.244.3.C182

[35] Hocking KM, Baudenbacher FJ, Putumbaka G, Venkatraman S, Cheung-Flynn J, et al (2013) Role of cyclic nucleotide-dependent actin cytoskeletal dynamics: [ca(2+)](i) and force suppression in forskolin-pretreated porcine coronary arteries. PloS one 8: e60986 10.1371/journal.pone.0060986 23593369 PMC3625185

[36] Hamachi T, Hirata M, Koga T (1984) Effect of cAMP-elevating drugs on Ca2+ efflux and actin polymerization in peritoneal macrophages stimulated with N-formyl chemotactic peptide. Biochimica et biophysica acta 804: 230–236. 6326851 10.1016/0167-4889(84)90154-x

[37] Hocking KM, Baudenbacher FJ, Putumbaka G, Venkatraman S, Cheung-Flynn J, et al (2013) Role of cyclic nucleotide-dependent actin cytoskeletal dynamics:Ca(2+)](i) and force suppression in forskolin-pretreated porcine coronary arteries. PloS one 8: e60986 10.1371/journal.pone.0060986 23593369 PMC3625185

[38] Woodrum D, Pipkin W, Tessier D, Komalavilas P, Brophy CM (2003) Phosphorylation of the heat shock-related protein, HSP20, mediates cyclic nucleotide-dependent relaxation. Journal of vascular surgery 37: 874–881. 12663991 10.1067/mva.2003.153

[39] Ba M, Singer CA, Tyagi M, Brophy C, Baker JE, et al (2009) HSP20 phosphorylation and airway smooth muscle relaxation. Cell health and cytoskeleton 2009: 27–42. 21686039 10.2147/chc.s5783 PMC3113651

[40] Rembold CM, Foster DB, Strauss JD, Wingard CJ, Eyk JE (2000) cGMP-mediated phosphorylation of heat shock protein 20 may cause smooth muscle relaxation without myosin light chain dephosphorylation in swine carotid artery. The Journal of physiology 524 Pt 3: 865–878. 10790164 10.1111/j.1469-7793.2000.00865.x PMC2269896

[41] Komalavilas P, Penn RB, Flynn CR, Thresher J, Lopes LB, et al (2008) The small heat shock-related protein, HSP20, is a cAMP-dependent protein kinase substrate that is involved in airway smooth muscle relaxation. American journal of physiology Lung cellular and molecular physiology 294: L69–78. 17993590 10.1152/ajplung.00235.2007 PMC2757925

[42] Dreiza CM, Brophy CM, Komalavilas P, Furnish EJ, Joshi L, et al (2005) Transducible heat shock protein 20 (HSP20) phosphopeptide alters cytoskeletal dynamics. FASEB journal: official publication of the Federation of American Societies for Experimental Biology 19: 261–263. 15598710 10.1096/fj.04-2911fje

[43] Chernik IS, Seit-Nebi AS, Marston SB, Gusev NB (2007) Small heat shock protein Hsp20 (HspB6) as a partner of 14-3-3gamma. Molecular and cellular biochemistry 295: 9–17. 17109079 10.1007/s11010-006-9266-8

[44] Gerthoffer WT (2005) Actin cytoskeletal dynamics in smooth muscle contraction. Canadian journal of physiology and pharmacology 83: 851–856. 16333356 10.1139/y05-088

[45] Rembold CM, Foster DB, Strauss JD, Wingard CJ, Eyk JE (2000) cGMP-mediated phosphorylation of heat shock protein 20 may cause smooth muscle relaxation without myosin light chain dephosphorylation in swine carotid artery. J Physiol 524 Pt 3: 865–878. 10790164 10.1111/j.1469-7793.2000.00865.x PMC2269896

[46] Campos-Bedolla P, Vargas MH, Calixto E, Segura P, Mendoza-Patino N, et al (2006) Alpha-methyl-5-HT, a 5-HT2 receptor agonist, stimulates beta2-adrenoceptors in guinea pig airway smooth muscle. Pharmacological research: the official journal of the Italian Pharmacological Society 54: 468–473. 10.1016/j.phrs.2006.09.006 17079161

[47] Harbeck B, Huttelmaier S, Schluter K, Jockusch BM, Illenberger S (2000) Phosphorylation of the vasodilator-stimulated phosphoprotein regulates its interaction with actin. J Biol Chem 275: 30817–30825. 10882740 10.1074/jbc.M005066200

[48] Kim HR, Graceffa P, Ferron F, Gallant C, Boczkowska M, et al (2010) Actin polymerization in differentiated vascular smooth muscle cells requires vasodilator-stimulated phosphoprotein. American journal of physiology Cell physiology 298: C559–571. 10.1152/ajpcell.00431.2009 20018948 PMC2838578

[49] Hocking KM, Brophy C, Rizvi SZ, Komalavilas P, Eagle S, et al (2011) Detrimental effects of mechanical stretch on smooth muscle function in saphenous veins. J Vasc Surg 53: 454–460. 10.1016/j.jvs.2010.09.010 21146345 PMC3053010

[50] Khalil RA, Crews JK, Novak J, Kassab S, Granger JP (1998) Enhanced vascular reactivity during inhibition of nitric oxide synthesis in pregnant rats. Hypertension 31: 1065–1069. 9576115 10.1161/01.hyp.31.5.1065

[51] Barany K, Rokolya A, Barany M (1990) Stretch activates myosin light chain kinase in arterial smooth muscle. Biochem Biophys Res Commun 173: 164–171. 2256911 10.1016/s0006-291x(05)81036-8

[52] Persechini A, Kamm KE, Stull JT (1986) Different phosphorylated forms of myosin in contracting tracheal smooth muscle. J Biol Chem 261: 6293–6299. 3516992

[53] Komalavilas P, Mehta S, Wingard CJ, Dransfield DT, Bhalla J, et al (2001) PI3-kinase/Akt modulates vascular smooth muscle tone via cAMP signaling pathways. J Appl Physiol 91: 1819–1827. 11568168 10.1152/jappl.2001.91.4.1819

[54] Gunst SJ, Zhang W (2008) Actin cytoskeletal dynamics in smooth muscle: a new paradigm for the regulation of smooth muscle contraction. Am J Physiol Cell Physiol 295: C576–587. 10.1152/ajpcell.00253.2008 18596210 PMC2544441

[55] Beall AC, Kato K, Goldenring JR, Rasmussen H, Brophy CM (1997) Cyclic nucleotide-dependent vasorelaxation is associated with the phosphorylation of a small heat shock-related protein. J Biol Chem 272: 11283–11287. 9111032 10.1074/jbc.272.17.11283

[56] Lopes LB, Brophy CM, Flynn CR, Yi Z, Bowen BP, et al (2010) A novel cell permeant peptide inhibitor of MAPKAP kinase II inhibits intimal hyperplasia in a human saphenous vein organ culture model. Journal of vascular surgery 52: 1596–1607. 10.1016/j.jvs.2010.06.168 20864298 PMC3005888

[57] Osgood MJ, Hocking KM, Voskresensky IV, Li FD, Komalavilas P, et al (2013) Surgical vein graft preparation promotes cellular dysfunction, oxidative stress, and intimal hyperplasia in human saphenous vein. Journal of vascular surgery. 10.1016/j.jvs.2013.06.004 PMC3926896 23911244

[58] Ba M, Singer CA, Tyagi M, Brophy C, Baker JE, et al (2009) HSP20 phosphorylation and airway smooth muscle relaxation. Cell Health and Cytoskeleton 1: 27–42. 10.2147/chc.s5783 PMC3113651 21686039

[59] Dreiza CM, Brophy CM, Komalavilas P, Furnish EJ, Joshi L, et al (2004) Transducible heat shock protein 20 (HSP20) phosphopeptide alters cytoskeletal dynamics. FASEB J: 04-2911fje. 10.1096/fj.04-2911fje 15598710

[60] Sudnitsyna MV, Seit-Nebi AS, Gusev NB (2012) Cofilin weakly interacts with 14-3-3 and therefore can only indirectly participate in regulation of cell motility by small heat shock protein HspB6 (Hsp20). Archives of biochemistry and biophysics 521: 62–70. 10.1016/j.abb.2012.03.010 22450169

[61] Cai Y, Nagel DJ, Zhou Q, Cygnar KD, Zhao H, et al (2015) Role of cAMP-Phosphodiesterase 1C Signaling in Regulating Growth Factor Receptor Stability, Vascular Smooth Muscle Cell Growth, Migration, and Neointimal Hyperplasia. Circulation research 116: 1120–1132. 10.1161/CIRCRESAHA.116.304408 25608528 PMC4702253

[62] Devynck MA, Simon A, Pernollet MG, Chironi G, Gariepy J, et al (2004) Plasma cGMP and large artery remodeling in asymptomatic men. Hypertension 44: 919–923. 15466659 10.1161/01.HYP.0000145862.33770.e9

[63] Kudo FA, Kondo Y, Muto A, Miyazaki K, Dardik A, et al (2009) Cilostazol suppresses neointimal hyperplasia in canine vein grafts. Surgery today 39: 128–132. 10.1007/s00595-008-3819-2 19198990

[64] Yamamoto K, Onoda K, Sawada Y, Fujinaga K, Imanaka-Yoshida K, et al (2007) Locally applied cilostazol suppresses neointimal hyperplasia and medial thickening in a vein graft model. Annals of thoracic and cardiovascular surgery: official journal of the Association of Thoracic and Cardiovascular Surgeons of Asia 13: 322–330. 17954989

[65] Li FD, Eagle S, Brophy C, Hocking KM, Osgood M, et al (2014) Pressure control during preparation of saphenous veins. JAMA surgery 149: 655–662. 10.1001/jamasurg.2013.5067 24759942 PMC4102634

[66] Clowes AW (1993) Intimal Hyperplasia and Graft Failure. Cardiovascular Pathology 2: S179–S186.

